# Impact of Commonly Used Transplant Immunosuppressive Drugs on Human NK Cell Function Is Dependent upon Stimulation Condition

**DOI:** 10.1371/journal.pone.0060144

**Published:** 2013-03-21

**Authors:** Aislin C. Meehan, Nicole A. Mifsud, Thi H. O. Nguyen, Bronwyn J. Levvey, Greg I. Snell, Tom C. Kotsimbos, Glen P. Westall

**Affiliations:** 1 Department of Medicine, Monash University, Melbourne, Victoria, Australia; 2 Department of Allergy, Immunology and Respiratory Medicine, The Alfred Hospital, Melbourne, Victoria, Australia; Hospital Infantil Universitario Niño Jesús, Spain

## Abstract

Lung transplantation is a recognised treatment for patients with end stage pulmonary disease. Transplant recipients receive life-long administration of immunosuppressive drugs that target T cell mediated graft rejection. However little is known of the impact on NK cells, which have the potential to be alloreactive in response to HLA-mismatched ligands on the lung allograft and in doing so, may impact negatively on allograft survival. NK cells from 20 healthy controls were assessed in response to Cyclosporine A, Mycophenolic acid (MPA; active form of Mycophenolate mofetil) and Prednisolone at a range of concentrations. The impact of these clinically used immunosuppressive drugs on cytotoxicity (measured by CD107a expression), IFN-γ production and CFSE proliferation was assessed in response to various stimuli including MHC class-I negative cell lines, IL-2/IL-12 cytokines and PMA/Ionomycin. Treatment with MPA and Prednisolone revealed significantly reduced CD107a expression in response to cell line stimulation. In comparison, addition of MPA and Cyclosporine A displayed reduced CD107a expression and IFN-γ production following PMA/Ionomycin stimulation. Diminished proliferation was observed in response to treatment with each drug. Additional functional inhibitors (LY294002, PD98059, Rottlerin, Rapamycin) were used to elucidate intracellular pathways of NK cell activation in response to stimulation with K562 or PMA-I. CD107a expression was significantly decreased with the addition of PD98059 following K562 stimulation. Similarly, CD107a expression significantly decreased following PMA-I stimulation with the addition of LY294002, PD98059 and Rottlerin. Ten lung transplant patients, not receiving immunosuppressive drugs pre-transplant, were assessed for longitudinal changes post-transplant in relation to the administration of immunosuppressive drugs. Individual patient dynamics revealed different longitudinal patterns of NK cell function post-transplantation. These results provide mechanistic insights into pathways of NK cell activation and show commonly administered transplant immunosuppression agents and clinical rejection/infection events have differential effects on NK cell function that may impact the immune response following lung transplantation.

## Introduction

Lung transplantation is an established treatment for patients with end stage pulmonary disease. Whilst lung transplant recipients (LTR) require life-long administration of immunosuppressive drugs to minimize alloreactivity and maintain optimal lung allograft function, episodes of acute cellular rejection remain relatively common and complications of chronic rejection and decline in lung function continue to impact on long term survival. LTR receive immunosuppressive drugs that target alloreactive T cells, the primary driver of acute cellular rejection. However, human studies suggest that other effector cells of the immune system, such as NK cells, may also have alloreactive potential and influence clinical outcomes following transplantation [1].

NK cells are a key component of the innate immune system, mediating cell lysis without prior antigen stimulation and were initially described as providing the first line of defence against tumours and viral infections. Whilst the intrinsic role of NK cells relates to host defence, more recent attention has focused on their role in influencing adverse clinical outcomes following allogeneic transplantation in the setting of either hematopoietic stem cells or solid organs [2], [3], [4], [5], [6].

Activation of NK cells is regulated by the balance between expressed inhibitory and activating NK cell receptors and their respective ligands on target cells [7]. These ligands typically include self HLA molecules. NK cells responding to HLA-mismatched ligands on the lung allograft have the potential to, both directly via engagement of receptor ligands on the allograft and indirectly through release of cytokines, enhance effector T cell activation and contribute to alloreactivity [8].

Following lung transplantation, an immunosuppressive regimen consisting of a calcineurin inhibitor, an anti-proliferative agent and a corticosteroid are given to suppress the immune response to the non-self allograft thereby minimizing episodes of rejection. Calcineurin inhibitors, such as Cyclosporine A or Tacrolimus, block the calcineurin pathway by forming complexes with cyclophilin and FK-binding protein, respectively. These immunophilins prevent calcineurin from dephosphorylating the NFAT transcription factor thus inhibiting transcription of genes encoding IL-2 and leading to a dampened effector T cell response [9]. Anti-proliferative agents including Azathioprine and Mycophenolate mofetil (MMF) impede lymphocyte growth and expansion. The anti-metabolite MMF is rapidly converted into its active form of Mycophenolic acid (MPA) after administration which then inhibits the enzyme, inosine monophosphate dehydrogenase, involved in *de novo* purine synthesis resulting in diminished lymphocyte proliferation [9], [10], [11]. Corticosteroids, such as Prednisolone, bind with glucocorticoid receptors, forming a complex which interacts with cellular DNA in the nucleus to modify gene transcription. Steroids impinge on various stages of antigen presentation, cytokine production and proliferation, all of which contribute to an anti-inflammatory and immunosuppressive effect [12], [13].

Given that there is little reported evidence relating to the impact of lung transplantation immunosuppressive drugs on NK cell function in either immunocompetent individuals or immunosuppressed lung transplant recipients (LTR), we performed a detailed analysis of the impact of a series of functional inhibitors on NK cell activity in healthy controls. These included clinically used immunosuppressive drugs such as a calcineurin inhibitor (Cyclosporine A), an anti-proliferative agent (MPA) and a corticosteroid (Prednisolone), but also the additional intracellular signalling inhibitor drugs Rapamycin (inhibitor of mTOR), Rottlerin (inhibitor of PKC in the NFkB pathway), LY294002 (inhibitor of Pi3K activity) and PD98059 (inhibitor of MEK in MAPK pathway). In addition, we studied NK cell function longitudinally both pre- and post- lung transplantation in a cohort of patients receiving immunosuppressive drugs.

## Materials and Methods

### Ethics Statement

All patients and controls gave written informed consent and the study was approved by The Alfred Hospital ethics committee (Project 175/02).

### LTR demographics and Controls

A group of 20 healthy volunteer controls, age and gender-matched to the LTR cohort, were recruited and analysed at a single time point. A cohort of 10 patients (mean age 41) who received HLA-mismatched bilateral lung transplants at The Alfred Hospital in 2009, were enrolled in a longitudinal analysis of NK cell function in response to immunosuppression. Individual LTR were identified based on disease status indicating the absence of immunosuppressive drugs prior to receiving a lung allograft. All LTR received a standard triple-therapy immunosuppression drug regime consisting of a calcineurin inhibitor (Tacrolimus), an anti-proliferative agent (Azathioprine) and a corticosteroid (Prednisolone). Induction therapy with the anti-thymocyte globulin (ATG) was given to two patients. LTR at-risk for CMV infection or reactivation (donor and/or recipient seropositive for CMV) were given intravenous ganciclovir (5 mg/kg) for 2 weeks followed by oral valganciclovir (900 mg/day) for a further 18 weeks. Surveillance bronchoscopy was performed at 1, 3, 6, 9 and 12 months post-transplantation or if clinically indicated, with bronchoalveolar lavage (BAL) and transbronchial biopsy sampling. Acute allograft rejection was diagnosed on transbronchial biopsy according to the International Society of Heart and Lung Transplantation guidelines [14]. At the time of routine surveillance bronchoscopy, whole blood samples (9 mL in sodium heparin tubes) from LTR were collected for later analysis of NK cell function.

### Cell preparation

Peripheral blood mononuclear cells (PBMC) were isolated from whole blood samples using Ficoll-Paque (GE Healthcare, NSW, Australia) and resuspended in RPMI-1640 containing 10% heat-inactivated FCS (SAFC, Sigma-Aldrich, NSW, Australia), 2 mM L-glutamine (GIBCO, NY, USA), 2 mM MEM non-essential amino acids (GIBCO), 100 mM HEPES (GIBCO), 50 µM 2-ME (GIBCO) and 1 U/ml penicillin/streptomycin (GIBCO); hereafter referred to as RF-10. PBMC thawed from cryopreserved LTR samples were rested overnight in 4 mL autologous plasma (diluted 1∶2 in RPMI-1640) prior to use in functional assays. The HLA class I negative target cell lines K562 and 721.221 were maintained in RF-10 media (approx 2.5×10^5^ cells/ml).

### Functional assessment of NK cell cytotoxic potential and cytokine production

Monoclonal antibodies (mAb) anti-CD3-PerCPCy5.5 (clone SK7) and anti-CD56-APC (clone NCAM 16.2) were used to phenotype both NK cell (CD56^+^CD3^−^) and T cell (CD56^−^CD3^+^) subsets, detected on a FACS Calibur flow cytometer (Becton Dickinson [BD], CA, USA). PBMC were stimulated with K562 target cells at a 2∶1 ratio for 6 h (37°C, 5% CO_2_). PMA (40 ng/ml, Sigma) with Ionomycin (1 μg/ml, Sigma), hereafter referred to as PMA-I, stimulation of PBMC was used as the positive control and unstimulated PBMC as the negative control. Anti-CD107a FITC (1∶20 dilution, clone H4A3) and Brefeldin A (10 μg/ml, Sigma) with monensin (2 µM, Sigma) were added to the cell culture at 0 and 1h, respectively. Cells were stained with anti-CD56 APC and anti-CD3 PerCPCy5.5 mAbs to differentiate NK cells and T cells, fixed (1% paraformaldehyde, ProSciTech, QLD, Australia), permeabilized (0.3% Saponin, Sigma) and stained with mAbs to detect production of intracellular IFN-γ PE (clone B27). All mAbs were purchased from BD Pharmingen and titrated to determine optimal staining. Samples were rested overnight at 4°C prior to performing flow cytometry. Lymphocytes were identified based on size and granularity. Both NK cell and T cell frequencies were then defined as a percentage of total lymphocytes and CD107a and IFN-γ positive expression defined as a percentage of total NK or T cells. Data was analysed using FlowJo software (TreeStar Inc, OR, USA).

### Immunosuppressive drugs and NK cell intracellular pathway functional assays

The impact of the commonly administered lung transplant immunosuppressive drugs Cyclosporine A (Novartis, NSW, Australia), MMF (using the active metabolite MPA; Sigma) and Prednisolone (Pfizer, NSW, Australia) on NK cell function was determined. Whilst the LTR received Tacrolimus and Azathioprine, these drugs are relatively unstable following prolonged storage and were unsuitable for the *in vitro* cultures performed, thus the alternative calcineurin inhibitor, Cyclosporine A, and anti-proliferative drug, MPA, were used in the assays performed with the healthy controls. Administered concentrations of the drugs were consistent with previous studies [12], [13], [15], [16], [17], [18] with each immunosuppressive drug being added to the NK cell and T cell functional assays at concentrations of 10 ng/ml, 100 ng/ml and 1000 ng/ml. These concentrations encompass physiologically equivalent standard therapeutic doses given to patients following transplantation. Drugs to inhibit intracellular signaling pathways were tested at a range of concentrations to determine toxicity to PBMC. Propidium iodide (PI) uptake by non-viable cells in response to inhibitor concentrations of LY294002 (Merck, VIC, Australia) and PD98059 (Merck) (each at 5 µM, 10 µM, 25 µM, 50 µM, 100 µM), Rapamycin (Merck; 10 nM, 50 nM, 100 nM, 200 nM, 500 nM) and Rottlerin (Merck; 1 µM, 2 µM, 5 µM, 10 µM, 50 µM) was used to determine the inhibitor drug concentrations to use in the subsequent NK cell functional assays. NK cells from six healthy controls were stimulated with K562 target cells and PMA-I (CD107a intracellular cytokine staining described previously) with or without the additional intracellular signalling inhibitors LY294002 (25 uM), PD98059 (25 uM), Rapamycin (100 nM) and Rottlerin (5 uM).

### Chromium release cytotoxicity assay

A standard chromium release cytotoxicity assay was used to assess NK cells lysis of K562 target cells, as previously described [6]. Briefly, ^51^Cr-labelled targets (2×10^3^ cells/well) were incubated with PBMC at effector-to-target (E:T) ratios of 50∶1, 100∶1 and 200∶1. Spontaneous-release and maximal-release controls were evaluated by incubating target cells with RF-10 and 1% Triton-X, respectively. Cytotoxicity was calculated as % specific lysis  =  [(experimental release – spontaneous release)/(maximal release – spontaneous release)] ×100.

### NK cell purification from whole PBMC and proliferation assay

PBMC were depleted of monocytes by overnight culture in RF-10 media and subsequent retrieval of non-adherent cells. NK cells were then isolated from the PBMC by magnetic bead negative selection according to the manufacturer's instructions (Magnetic Activated Cell Sorting (MACS) NK cell isolation kit, Miltenyi Biotech, Teterow, Germany) to achieve a purity of greater than 98% CD56^+^ CD3^−^ NK cells. MACS enriched NK cells, from three of the 20 controls, were labelled with 1 µM CFSE (Sigma) at a cell density of 10^7^/ml in PBS. After 5 min at 37°C, 5% CO_2_, cells were washed once with PBS containing 1% FCS, washed once with PBS containing 0.1% FCS and resuspended in RF-10. CFSE labelled NK cells were plated in triplicate into 96 well U-bottom plates at 5×10^4^ cells/well for three days of *in vitro* culture at 37°C, 5% CO_2_. NK cells were stimulated to proliferate with the addition of the cell line 721.221 at a 1∶1 ratio and a combination of IL-2 (250 U/ml; Peprotech, NJ, USA) and IL-12 (10 U/ml; Peprotech) cytokines in the presence or absence of immunosuppressive drugs. Both media and immunosuppressive drugs were replenished every second day. Cell staining, acquisition and analysis of NK cells was performed as described in the previous section.

### Statistical analysis

Numerical data were expressed as means ± standard of error (SEM). Repeated one-way analysis of variance (ANOVA) was used to assess differences in NK cell CD107a and IFN-γ expression and proliferation at each concentration of drug used. One-way ANOVA was performed to assess differences between pre- and post-transplant NK cell function compared to healthy controls. Statistical significance was defined as *p*<0.05 using GraphPad Prism version 5.00 for Windows (GraphPad Software, San Diego, CA, USA).

## Results

### Differential effect of immunosuppressive drugs on NK cell cytotoxicity is stimulus dependent *in vitro*

**Figure 1:**
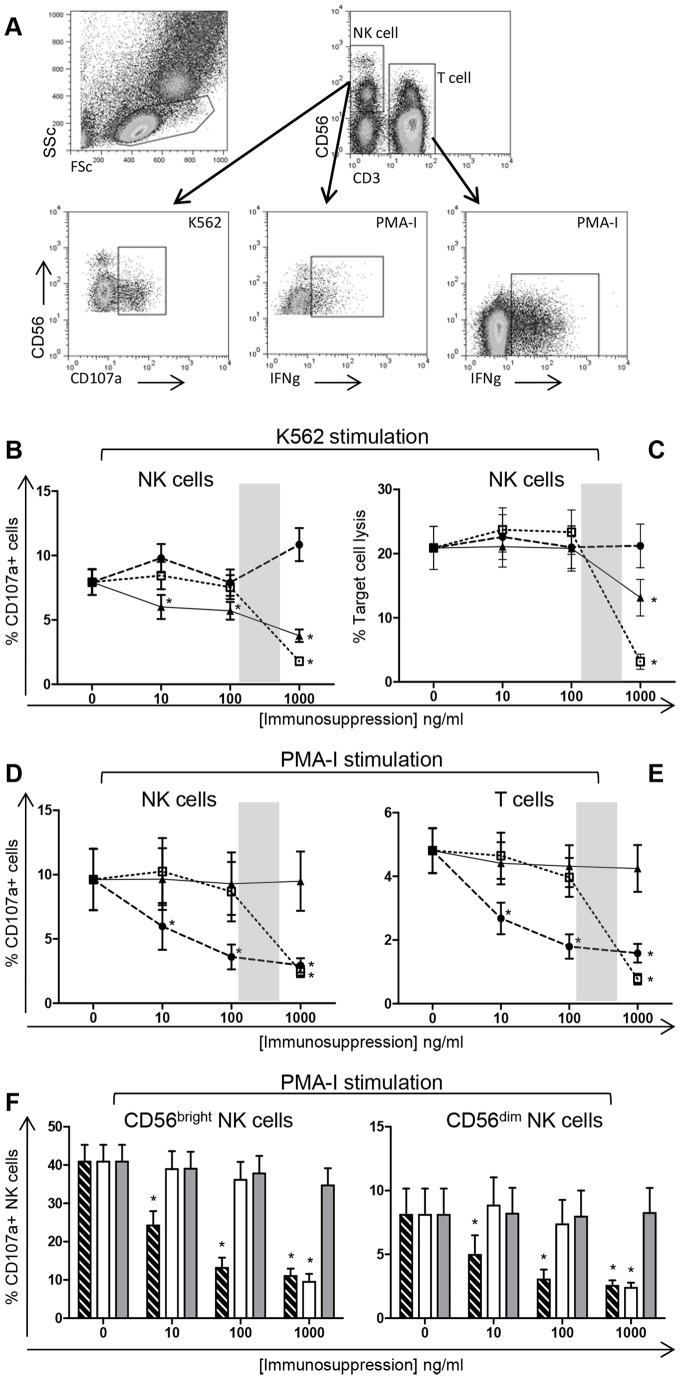
NK cell and T cell cytotoxicity in the presence of immunosuppressive drugs. PBMC from 20 healthy controls were stimulated in culture with the cell line K562 or PMA-I in the presence or absence of varying concentrations of immunosuppressive drugs. An example of the flow cytometry gating strategy for identification of positive expression is shown (A). NK cell cytotoxicity measured by CD107a surface expression (B) and chromium release assay, at a 50∶1 effector-to-target ratio (C), in response to K562 stimulation. CD107a expression for whole CD56+ NK cells (D), T cells (E) and NK cell subsets CD56bright and CD56dim (F) measured following PMA-I stimulation. Statistical significance was defined as *p*<0.001. Graphed data are presented as mean ± SEM. Symbols represent immunosuppressive drugs: •, dashed bars: Cyclosporine A; □, white bars: MPA; ▴, grey bars: Prednisolone. Shaded areas signify therapeutic range.

To determine the cytotoxic potential of activated NK cells, cell surface expression of CD107a indicating recent degranulation of cytotoxic granules, was used as the surrogate marker (Figure 1A) [19], [20]. Compared to baseline CD107a expression in the absence of immunosuppressive drugs (7.9%±1.0%), there was a dose-response decline with the addition of Prednisolone at 10 ng/ml (6.0%±0.9%), 100 ng/ml (5.7%±0.7%) and 1000 ng/ml (3.8%±0.5%). Whereas, only high dose MPA significantly reduced CD107a expression (1.8%±0.3%) and no effect was observed for Cyclosporine A treatment (Figure 1B).

To support these findings, a standard chromium release assay was used as an alternate measure of NK cell cytotoxicity. The kinetics mirrored those observed in the CD107a cell surface expression assay for treatment with MPA and Cyclosporine A. However, in this assay system only addition of high dose Prednisolone had a significant effect compared to the control (13.1%±2.9% vs 20.9%±3.3%) (Figure 1C). The chromium release assay system was found to be less sensitive than the flow cytometry based CD107a assay which was able to identify more subtle changes in NK cell cytotoxicity.

Stimulation of PBMC was also achieved using PMA-I. Surprisingly, inverse kinetic profiles for both Cyclosporine A and Prednisolone were shown, compared to that observed using K562 cell line as the stimulus. In contrast to baseline NK cell (9.6%±2.4%) and T cell (4.8%±0.7%) expression, Cyclosporine A significantly reduced CD107a expression at 10 ng/ml (6.0%±1.8% and 2.7%±0.5%), 100 ng/ml (3.6%±1.0% and 1.8%±0.4%) and 1000 ng/ml (2.9%±0.5% and 1.6%±0.3%), respectively. High dose MPA decreased CD107a expression on NK cells (2.5%±0.4%) and T cells (0.8%±0.2%) whilst Prednisolone demonstrated no change (Figures 1D, E).

When the whole NK cell population was analysed into the two main subsets of CD56bright and CD56dim NK cells, it was observed that the function of both NK cell subsets was influenced in the same way with the addition of immunosuppression, thus data was presented as whole CD56+ NK cells. Although following PMA-I stimulation, but not with K562 stimulation, the decline in positive expression of CD107a was more striking in the CD56bright subset compared to the CD56dim cells suggesting the CD56bright cells were more severely affected by the immunosuppressive drugs (Figure 1F).

### Clear link between cytokine production and cytotoxicity profiles in NK cells

**Figure 2:**
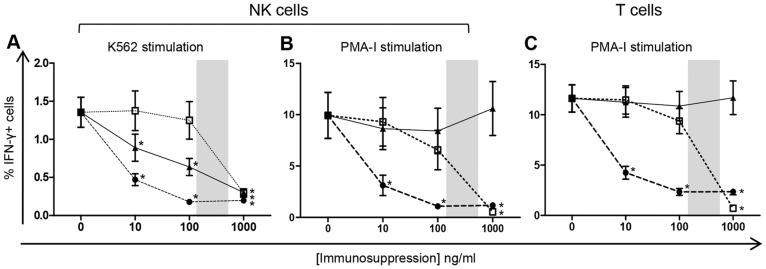
NK cell and T cell IFN-γ production in the presence of immunosuppressive drugs. PBMC from 20 healthy controls were stimulated in culture with the cell line K562 or PMA-I in the presence of varying concentrations of immunosuppressive drugs. NK cell IFN-γ production measured in response to stimulation with K562 cell line (A) and PMA-I (B). T cell IFN-γ production measured in response to PMA-I stimulation (C). Statistical significance was defined as *p*<0.001. Graphed data are presented as mean ± SEM. Symbols represent immunosuppressive drugs: •, Cyclosporine A; □, MPA; ▴, Prednisolone. Shaded areas signify therapeutic range.

The impact of immunosuppression on NK cell activation was evaluated by quantitative measurement of IFN-γ cytokine production (Figure 1A). In the absence of immunosuppression the percentage of NK cells producing IFN-γ was 1.3%±0.2% and 9.9%±2.2% following stimulation with either K562 cell line or PMA-I, respectively (Figures 2A, B). Interestingly, IFN-γ cytokine profiles were similar to those of cytotoxicity with K562 stimulation, showing dose-response decreases were shown with addition of Cyclosporine A at 10 ng/ml (0.5%±0.08%), 100 ng/ml (0.2%±0.02%) and 1000 ng/ml (0.2%±0.02%) and Prednisolone at 10 ng/ml (0.9%±0.2%), 100 ng/ml (0.6%±0.1%) and 1000 ng/ml (0.3%±0.04%), whilst MPA significantly decreased IFN-γ production only at the highest concentration (0.3%±0.06%) (Figure 2A).

Stimulation of both NK cells and T cells with PMA-I emulated data obtained in the cytotoxicity assays. High dose MPA significantly reduced IFN-γ production by NK cells (0.54%±0.09%) and T cells (0.7%±0.1%). Cyclosporine A significantly reduced IFN-γ production by NK cells and T cells at 10 ng/ml (3.1%±1.0% and 4.3%±0.6%), 100 ng/ml (1.1% ±0.2% and 2.3%±0.3%) and 1000 ng/ml (1.2%±0.2% and 2.4%±0.3%), respectively (Figures 2B, C). The same effect, as that observed with CD107a, was also seen for IFN-γ expression by CD56bright and CD56dim NK cells (data not shown).

### NK cell proliferation impeded by use of clinical immunosuppressive drugs

**Figure 3:**
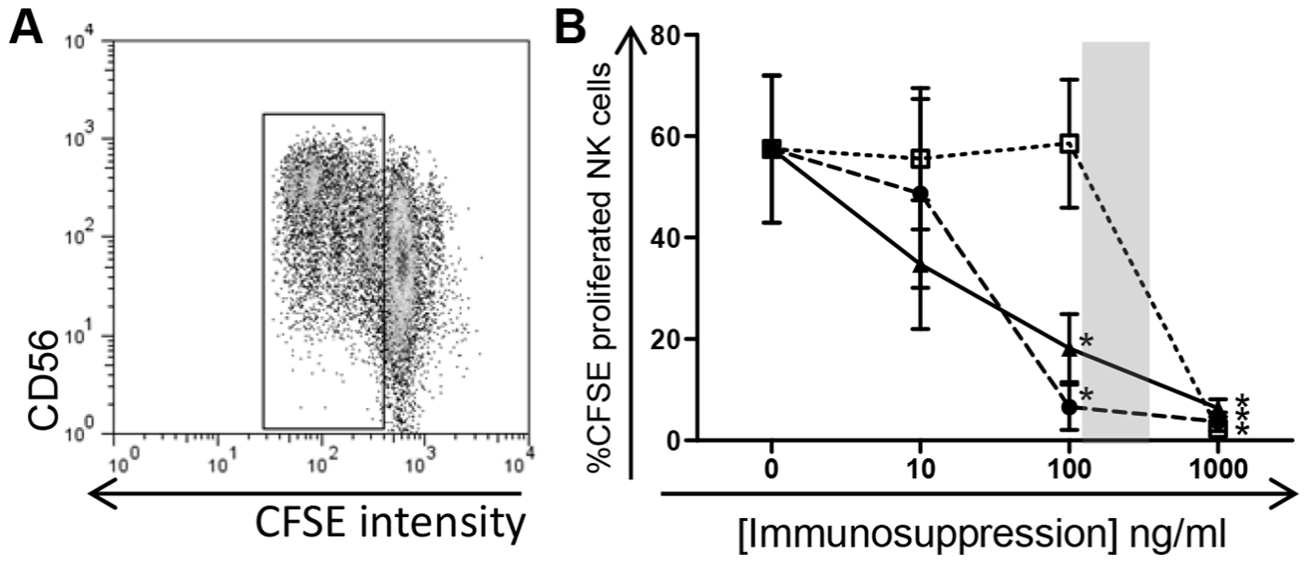
Proliferation of NK cells in the presence of immunosuppressive drugs. MACS enriched NK cells from three healthy controls were labelled with CFSE and stimulated in culture for three days with a combination of IL-2, IL-12 and 721.221 cell lines in the presence or absence of immunosuppressants Cyclosporine A, MPA and Prednisolone. An example of the change in CFSE intensity as the cells proliferate is shown (A). NK cell proliferation is displayed in response to treatment with varying concentrations of the immunosuppressive drugs (B, *p*<0.05 for all). Graphed data are presented as the mean ± SEM from three independent experiments. Symbols represent immunosuppressive drugs: •, Cyclosporine A; □, MPA; ▴, Prednisolone. Shaded area signifies therapeutic range.

The ability of NK cells to proliferate *in vitro* following stimulation with the 721.221 cell line with IL-2 and IL-12 cytokines was measured by decreased CFSE intensity compared to the undivided parental population (Figure 3A). The dynamics of proliferating NK cells under immunosuppressive conditions matched those of cytokine production with a significant decline in proliferation from baseline (57.5%±14.5%) in the presence of Cyclosporine A at 100 ng/ml (6.6%±4.5%) and 1000 ng/ml (3.7%±1.8%), Prednisolone at 100 ng/ml (18.2%±6.7%) and 1000 ng/ml (6.3%±1.7%) and MPA at 1000 ng/ml (2.3%±0.8%) (Figure 3B).

### Additional inhibition of intracellular signalling pathways

**Figure 4:**
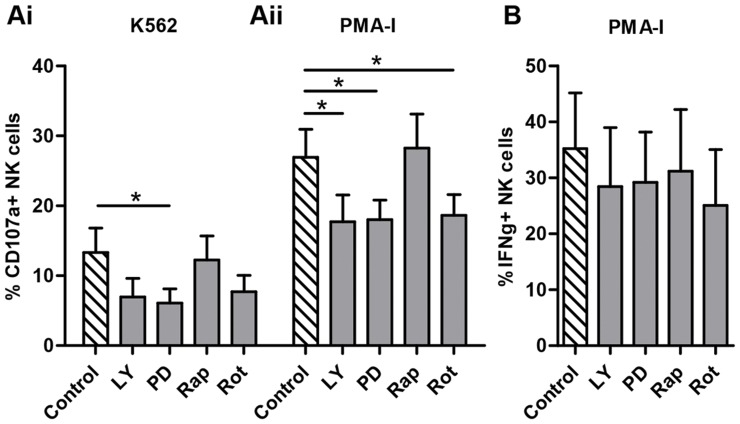
Inhibition of intracellular signaling pathways. NK cells from six controls were cultured with K562 and PMA-I alone (control) or in the presence of the inhibitor drugs LY294002 (25 µM), PD98059 (25 µM), Rapamycin (100 nM) and Rottlerin (5 µM). NK cell function was assessed for CD107a expression (A) and IFN-γ production (B) in response to addition of inhibitor drugs in culture. CD107a expression is shown to be reduced by addition of inhibitor drugs affecting pathways of cellular activation. Statistical significance was defined as *p*<0.05. Graphed data presented as mean ± SEM.

To help elucidate which intracellular signalling pathways the K562 cell line and PMA-I stimulation of NK cells were acting through, additional functional assays were performed with immune cell inhibitor drugs LY294002 (inhibitor of Pi3K activity), PD98059 (inhibitor of MEK in MAPK pathway), Rapamycin (inhibitor of mTOR) and Rottlerin (inhibitor of PKC in the NFkB pathway). The toxicity at five concentrations of each inhibitor was tested by determining PI uptake by non-viable cells as compared to the control. The percentage of PI+ non-viable cells was observed to decide the optimal non-toxic concentration to proceed with in further assays (data not shown). NK cells were stimulated with K562 target cells and PMA-I, as described previously, with or without the addition of the inhibitors LY294002 (25 uM), PD98059 (25 uM), Rapamycin (100 nM) and Rottlerin (5 uM). It was observed that CD107a expression after K562 stimulation was significantly decreased with the addition of the inhibitor PD98059 at 25 uM (13.3%±3.5% vs 6.1%±2.0%), whilst there was a trend for a decline observed with LY294002 and Rottlerin (Figure 4Ai). Similarly, following PMA-I stimulation there was a significant decrease in CD107a expression compared to the control (27.0%±4.0%) with the addition of LY294002 (17.7%±3.8%), PD98059 (18.0%±2.8%) and Rottlerin (18.6%±3.0%) (Figure 4Aii). No effect was observed on IFNγ production following PMA-I stimulation (Figure 4B).

### Lung transplantation affects NK cell function

**Figure 5:**
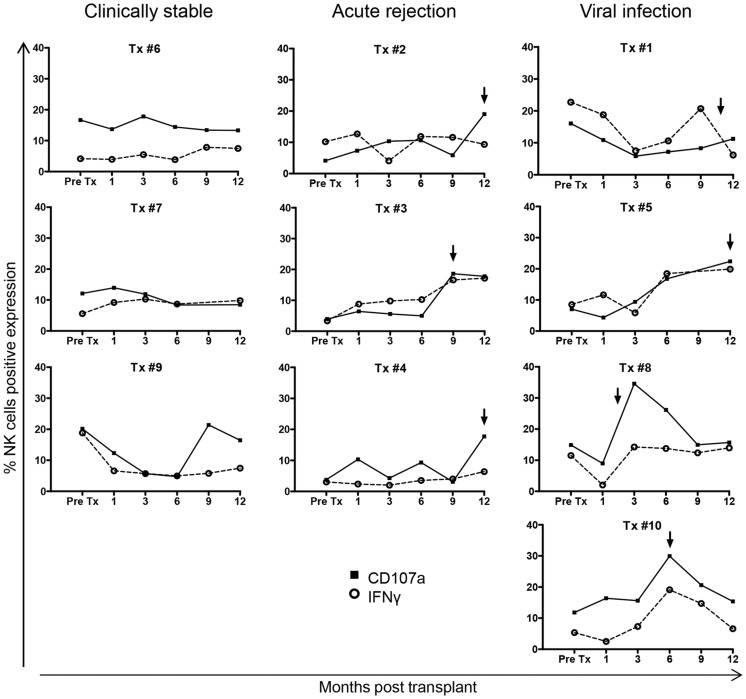
Functional changes in NK cells from lung transplant patients. PBMC from ten LTR, not receiving immunosuppression pre-transplant (Pre-Tx), were stimulated in culture with PMA-I. Individual graphs of NK cell CD107a expression (closed square) and IFN-γ production (open circle) from LTR with clinically stable post-transplantation follow-up (Tx #6, #7, #9), episodes of acute rejection (Tx #2, #3, #4) or viral infection (Tx #1, #5, #8, #10). The arrows represent the occurrence of each clinical event in the months post-transplant.

Ten LTR, all of whom were not receiving immunosuppression pre-transplant, were included in a longitudinal analysis of NK cell function in response to immunosuppression. Demographic details of the LTR are shown in Table 1. The expression of both CD107a and IFN-γ on NK cells following PMA-I stimulation did not significantly differ between healthy controls (n = 20) or transplant recipients group analyses, either pre-transplant or within the first year post-transplant (data not shown). However individual patient dynamics revealing three different longitudinal patterns of NK cell function were observed (Figure 5). Stable NK cell function over time was seen in two of three patients who in the first year post-transplant had no evidence of either allograft rejection or viral infection (clinically stable). Four patients who developed viral infections early post-transplant demonstrated a transient increase in NK cell cytotoxicity contemporaneous to the viral infection. The observed changes in NK cell activity were not found to be solely attributable to the induction therapy the patients (Tx#1 and Tx#8) received. Finally, increased NK cell cytotoxicity was seen in patients who developed histologically-confirmed acute cellular rejection between 9 and 12 months post-transplant. Compared to pre-transplant levels, the three patients who experienced acute cellular rejection demonstrated a 3.6 fold increase in NK cytotoxicity at the time of allograft rejection, whilst the four patients who developed viral infections demonstrated a 2.3 fold increase in NK cell cytotoxicity at the time of viral recrudescence compared to baseline values. Given that the study was limited to the first year post-transplant, we were unable to confirm that NK cell function decreased to basal levels following clinical intervention.

**Table 1:** Lung transplant patient and healthy control demographics.

Patient	Age at time of Tx	Gender	Transplantation Indication	CMV Serostatus D/R
Tx #1*	30	M	CF	−/−
Tx #2	58	M	EMP	+/+
Tx #3	30	F	CF	+/−
Tx #4	52	F	EMP	−/+
Tx #5	30	F	CF	−/−
Tx #6	30	M	CF	−/−
Tx #7	51	F	EMP	−/−
Tx #8*	59	M	COPD	+/+
Tx #9	52	M	EMP	−/−
Tx #10	19	M	BRON	−/+
Control #1–20	Mean: 42	10M/10F	*NA*	*NA*

Tx indicates transplant; D indicates donor; R indicates recipient; CF indicates cystic fibrosis; EMP indicates emphysema; COPD indicates chronic obstructive pulmonary disease; BRON indicates bronchiectasis; *NA* indicates not applicable.

* Patients who received Anti-thymocyte globulin.

## Discussion

In a cohort of healthy controls and LTR we performed a detailed analysis of NK cell function in the presence of differing stimulation conditions and following administration of the commonly used clinical immunosuppressants, calcineurin inhibitors, anti-proliferative agents and corticosteroids. We demonstrated using well-defined *in vitro* assays that the addition of specific immunosuppressive drugs differentially impacted on NK cell cytotoxicity, cytokine production and proliferation, which was dependent on the primary stimulus. Importantly, these immunosuppressive drugs were found to impair NK cell function at concentrations corresponding to the therapeutic range used in the management of lung transplant patients [9], [10], [21], [22], [23].

The absence of HLA class I molecules expressed on K562 means that there is no inhibitory signal provided when NK cell receptors engage with the target cells resulting in NK cell activation and the release of cytotoxic granules containing perforin and granzymes and subsequent target cell lysis [7]. Observation of the differential effects of each of the three immunosuppressive drugs (Cyclosporine A, MPA and Prednisolone) on inhibiting NK cell cytotoxity following K562 stimulation provides mechanistic insights into how these drugs influence NK cell activation pathways. Unlike MPA and Prednisolone, Cyclosporine A, an inhibitor that effectively reduces IL-2 production by blocking the calcineurin pathway [9], failed to inhibit NK cell cytotoxicity following K562 stimulation, suggesting activation of NK cells by K562 occurs via a calcineurin-independent pathway. Given that Cyclosporine A prevents calcineurin from dephosphorylating the NFAT transcription factor thus inhibiting transcription of genes encoding IL-2 and leading to a dampened effector T cell response, it is likely that NK cells have a similar intracellular calcineurin pathway to T cells. This finding of ineffectual activity towards NK cell cytotoxicity corroborates previous reports demonstrating that NK cells cultured in the presence of Cyclosporine A retained their cytotoxic capabilities against various target cell lines, including K562 [16], [18], [24], [25], [26]. Similarly, the alternative calcineurin inhibitor Tacrolimus was also found to have no effect on NK cell cytotoxicity against target cells [16], [27], [28]. However there are contradictory reports in the literature as to whether NK cell degranulation/cytotoxicity is affected by Cyclosporine [27], [29], [30]. The differences observed in these studies may be explained by the use of different experimental techniques used in these assays and pre-incubation of effector cells with immunosuppressive drugs in culture for an extended period of time which likely severely impairs the activity of the cells prior to testing in functional assays.

The effects of Prednisolone and MPA on decreasing NK cell cytotoxicity fits with published observations describing impaired NK cell lysis of K562 target cells in an *in vitro* environment [25], [31] down regulation of NK cell activating receptors and inhibition of cytotoxic granule exocytosis [12], [13]. With the inclusion of additional intracellular pathway inhibitors LY, PD and Rottlerin, NK cell function following K562 stimulation was shown to be decreased. However as this observation was only significantly affected with the addition of PD, it can be surmised that K562 stimulation of NK cell cytotoxicity has most influence acting through the MAP-kinase pathway of activation.

To determine whether the type of stimulus was a critical factor influencing the role of immunosuppressive drugs on NK cell function, PMA-I was used as an alternative stimulant. PMA is a phorbol ester that induces activation of Protein Kinase C (PKC), resulting in phosphorylation of activators of transcription leading to increased gene expression [32]. Ionomycin is an ionophore that raises the intracellular level of calcium and is commonly used in conjunction with PMA to stimulate intracellular production of various cytokines including interferons and IL-2 [32], [33]. Interestingly following PMA-I stimulation, an inverse inhibition pattern was observed in NK cells following treatment with Prednisolone and Cyclosporine A, compared to K562 stimulation. Specifically, high dose MPA and Cyclosporine A significantly reduced NK cell cytotoxicity, whilst Prednisolone had no functional effect. Given the mechanism of inhibition by Cyclosporine A in this setting, it is likely that NK cell activation following PMA-I stimulation is directed via the calcineurin pathway of intracellular signalling possibly in response to increased levels of calcium (which activate calcineurin) due to inclusion of Ionomycin. Cytotoxicity inhibition kinetics were shown to be identical for both NK cell and T cell populations, suggesting that the same intracellular signalling pathways are used following PMA-I stimulation. As the addition of the inhibitors LY, PD and Rottlerin caused a significant reduction in NK cell cytotoxicity in a similar manner to that observed with Cyclosporine and MPA, it can be suggested that the point of action of PMA-I on NK cell stimulation occurs through multiple pathways of intracellular activation (including PKC and calcineurin pathways, as expected) or alternatively at a common point downstream of each pathway.

The production of immunomodulatory cytokines following stimulation is a hallmark feature associated with functionally activated cells. Following K562 stimulation, the ability of NK cells to produce IFN-γ was severely impaired by individual treatment with the three immunosuppressive drugs, with NK cells exhibiting a 100-fold increase in sensitivity in the presence of Cyclosporine A and Prednisolone compared to MPA. However, this inhibition was not mirrored following use of the alternate stimulus PMA-I. In this setting NK cells treated with high does Prednisolone did not show impairment of IFN-γ production, which was also observed in the T cell population. Collectively, these observations convincingly demonstrated that immunosuppressive drugs have differential NK cell functional effects, which are dependent on the primary stimulus for the induction of independent intracellular signalling pathways. Cyclosporine A inhibits cytokine production via calcineurin inhibition. On the other hand, Prednisolone inhibition of NK cell function may be related to its action at the transcriptional level to regulate gene expression. Prednisolone has been shown to inhibit the expression of NK cell receptors necessary for activation and cytotoxicity [12], [13]. Depending on the NK cell stimulus used in our culture setting, either receptor engagement with target cell or through the action of PMA-I, different transcription factors are likely to be involved to promote different gene expression for various aspects of NK cell function.

In addition to investigating the ability of NK cells to induce cytotoxicity and cytokine production in the presence of immunosuppressive drug treatment, the proliferative capacity following dual stimulation with cytokines (IL-2 and IL-12) and a HLA class-I deficient cell line (721.221) was also explored. A dose-dependent reduction in NK cell proliferation for both Prednisolone and Cyclosporine A was observed with a 10-fold increase in sensitivity compared to MPA treatment. Interestingly, although we expected that the anti-proliferative agent, MPA, would have dramatically reduced NK cell proliferation at all concentrations tested this was not the case. This observation may be explained by the presence of IL-2 at a high concentration of 250 U/ml, a prominent stimulator of lymphocyte activation and growth, in the assay culture used to stimulate the NK cells [34], [35]. The addition of IL-2 may have provided an environment conducive to dampen down the immunosuppressive effects of MPA at lower concentrations but not influence the drug action of Cyclosporine A or Prednisolone. Cyclosporine A selectively blocks IL-2 transcription in activated cells and impairs both the synthesis and cell surface expression of the high affinity IL-2R [9]. Prednisolone, through its action of modifying gene transcription, may also act to reduce expression of the IL-2R thus causing NK cells to not become activated in response to the IL-2 in the culture medium. The absence of both IL-2 and the IL-2R would reduce the proliferative potential of NK cells following stimulation. The data generated in our *in vitro* assay system correlated with a previous report demonstrating reduced proliferation of the major blood population of CD56dim NK cells in response to Cyclosporine A [18] and impaired NK cell proliferation and survival with the addition of methylprednisolone following IL-2 stimulation [13]. As prolonged exposure to Cyclosporine A for 7 days and Prednisolone for 5 days has been shown to induce NK cell apoptosis [13], [18], and activation-induced cell death occurs in a proportion of NK cells after they bind to a target cell and carry out ‘natural cytotoxicity’ [36]; the observed loss in proliferation after three days culture may be influenced by the death of some NK cells.

We have previously published work showing changes in the phenotype of NK cells corresponding to clinical episodes in the early months following lung transplantation [6]. However this work did not directly address NK cell functional changes post-transplantation.

This longitudinal study follows on from the phenotypic analysis, to assess NK cell function in LTR and has revealed a number of interesting findings. Surprisingly NK cell function (cytotoxicity and IFN-γ production), as a collective group, did not significantly vary between normal controls and LTR when analysed cross-sectionally. Whilst NK cells might be expected to become activated following a HLA-mismatched transplant, this may have been nullified by the addition of immunosuppressive drugs. The longitudinal analysis showed that even in the presence of immunosuppressive drugs, NK cells are capable of becoming transiently more active in the setting of self-limiting viral infections or acute rejection. While this study was not able to assess whether NK cells are primarily responsible for viral clearance following infection, it is worth noting that viral infections in immunosuppressed individuals are associated with increased viral load suggesting that viral clearance mechanisms are impaired in this setting [37], [38].

This study showed that NK cell function is impaired in the presence of therapeutic doses of immunosuppressive drugs that are commonly given to solid organ transplant recipients. Our findings confirm other published results demonstrating altered NK cell function in response to transplantation immunosuppression and support the view that this is an important area for future investigation in both the laboratory and in the clinic. We demonstrated that NK cell function changed with time from transplant and in parallel to clinical episodes of viral infection and acute rejection. Future studies are now being undertaken to formally assess NK cell alloreactivity against KIR-ligand mismatched targets both in the presence and absence of commonly used immunosuppressive drugs, and as to whether NK cell profiles influence the long term survival of lung allografts following transplantation.
